# Liquid Embolization of Peripheral Arteriovenous Malformations with Ethylene-Vinyl Alcohol Copolymer in Neonates and Infants

**DOI:** 10.1155/2022/1022729

**Published:** 2022-07-18

**Authors:** Jochen Pfeifer, Walter A. Wohlgemuth, Hashim Abdul-Khaliq

**Affiliations:** ^1^Department of Pediatric Cardiology, Saarland University Medical Center, Homburg 66421, Germany; ^2^Department for Radiology, Martin-Luther-University Halle-Wittenberg, Halle 06120, Germany

## Abstract

In the postnatal period, extensive peripheral arteriovenous malformations (AVM) are associated with high morbidity, especially when localized in the liver. Their urgent treatment is always a challenging problem in neonates and infants. We analyzed four consecutive children aged three days to three years who underwent eight liquid embolization procedures with ethylene-vinyl alcohol copolymer. The AVM were situated on the thoracic wall, in the liver, and on the lower leg. In three cases, the malformations showed total regression. The tibial AVM degenerated widely. If impaired beforehand, cardiac or hepatic function normalized after the interventions. There were no embolization-associated complications such as nontarget embolization or tissue ischemia. We conclude that application of ethylene-vinyl alcohol copolymer seems to be a safe therapeutic option and can be used in neonates and infants with peripheral AVM in consideration of the agent's characteristics. Nevertheless, there are still hardly any data concerning young children.

## 1. Introduction

Vascular anomalies in children are rare congenital malformations. According to the International Society for the Study of Vascular Anomalies (ISSVA) [1, 2], they are divided into two subgroups: vascular tumors and vascular malformations. The most common type of vascular tumors are benign hemangiomas. Vascular malformations are classified into venous, capillary, lymphatic, arteriovenous, and combined malformations, depending on their main vessel structure and hemodynamics. Arteriovenous malformations (AVM) account for about 8% of all vascular malformations [3]. This type is characterized by irregular feeding arteries, shunting blood directly into a vein through a “nidus” consisting of arterial microfistulae [4, 5]. AVM are part of the high-flow vascular malformations. Peripheral AVM are located outside the central nervous system.

AVM tend to grow progressively. There are various clinical symptoms and manifestations of AVM depending on their dimension, localization, and shunt volume affecting the circulatory system. The previously published Schobinger score provides a clinical staging of AVM. These four clinical stages are quiescence (stage I; cutaneous blush or warmth), expansion (stage II; bruit or thrill, increasing size, pulsation, and no pain), local destruction (stage III; pain, bleeding, infection, skin necrosis, or ulceration), and decompensation (stage IV; high-output cardiac failure) [4, 6, 7]. In contrast, coagulopathy is rather found in slow-flow malformations [8].

If associated with severe systemic symptoms, extensive AVM require urgent treatment. The therapy of AVM in young children is always a challenging problem because of the patients' small size and the restricted adaptation of their circulatory system. The therapeutic strategy depends on the localization, size, and the clinical manifestation. Small AVM may be resected surgically, whereas resection is not suitable for extensive lesions due to higher perfusion by multiple feeding arteries and hence the higher risk for perioperative bleeding and recrudescence. Selective ligation or embolization of feeding arteries is not promising, for it leads to further angiogenesis without AVM involution. Conservative and medical treatment options have also been found inappropriate [7, 9].

Percutaneous and transvascular embolization are considered to be first line therapy in advanced state AVM regarding the Schobinger score [5, 7, 10]. For transcatheter use, various mechanical occlusion devices (coils, vascular plugs) and liquid embolic agents are available. The latter are divided into sclerosing agents (ethanol), polymerizing agents (cyanoacrylate or ethylene-vinyl alcohol copolymer = EVOH), and particulate agents [11]. To avoid relapse, complete occlusion of the vascular nidus has to be the goal of the embolization. Of note, exclusive embolization of single-feeding arteries has the same unsatisfactory effect as their surgical ligation. Therefore, implantation of coils or plugs is appropriate in addition to liquid embolization in selected cases [7].

EVOH is originally used in interventional neuroradiology. Besides, there are several published studies of successful embolization of peripheral AVM with EVOH primarily in adults [5, 9, 12] and children older than 3 years [13].

The aim of our retrospective study was to present our experience in urgent AVM embolization using EVOH in neonates and infants in different localizations.

## 2. Material and Methods

### 2.1. Patients

We performed 8 liquid embolization procedures in 4 consecutive children, two of them male. The parents gave written informed consent the day before the intervention.

Two AVM were located on the thoracic wall, one was a pretibial AVM; and there was one newborn with prenatal diagnosis of huge intrahepatic AVM. The patients' characteristics are summarized in Table 1.

Patient 1 had undergone a surgical correction of atrioventricular septal defect at the age of 5 months. A thoracic AVM showed increasing size (finally 7 × 5 × 2.5 cm) with increasing flowing shunt. During follow-up, the arteriovenous shunt was associated with progressive cardiac output and increasing mitral regurgitation, and cardiomegaly occurred. Neither anticongestive treatment with propranolol nor coil occlusion of feeding arteries deriving from the subclavian artery with 5 coils (MReye©Flipper© detachable coils, Cook© medical, Bloomington, Indiana, USA) were successful to induce significant reduction of the AVM perfusion, since further feeding arteries were also established from the intercostal arteries. Liquid embolization was then indicated and successfully performed.

Patient 2 presented with a growing AVM (9 × 8 × 2.5 cm) of the thoracic wall. At the age of 6 months, he was admitted to the emergency department with a severe superficial bleeding due to accidental skin erosions covering the AVM. Emergency hemostasis was achieved by Neodym-Yag laser treatment and compression dressings. After stabilization and healing of the superficial skin erosions, we decided to subject the child to transcatheter embolization using EVOH.

Patient 3 had a growing pretibial AVM (7 × 4 × 4.2 cm) on the right leg with tight consistency. Embolization was indicated because of imminent rupture due to hyperperfusion of the AVM.

In patient 4, AVM and cardiomegaly were diagnosed prenatally. The major part of the AVM was located in the liver segments 2 and 3. Giant veins drained into the lower vena cava. After birth, the newborn was critically ill because of severe high-output heart failure and pulmonary hypertension. Highly urgent embolization therapy was indicated.

### 2.2. Transcatheter Embolization

All procedures were performed by percutaneous transarterial catheterization via the right femoral artery. After puncture, 4 French (Fr) introducers were inserted in each case. 4 Fr guiding catheters were used for angiography and guiding of microcatheters. In the case of the pretibial AVM, the sheath was introduced antegrade into the femoral artery. As antithrombotic therapy, heparin was administered in a dose of 100 IU per kilogram bodyweight.

EVOH (Onyx©, EV3, Irvine, California, USA) was used for liquid embolization. This agent is an ethylene-vinyl alcohol copolymer, which is blended with radiopaque tantalum and dissolved in dimethyl sulfoxide (DMSO). In contact with other aqueous fluids such as water or blood, EVOH precipitates by polymerization and hardens from the surface to the inside. It hence builds an occlusive cast, when intravascularly applied. EVOH is available in 3 concentrations with increasing viscosity: Onyx©-18, -20, and -34, containing 6%, 6.5%, and 8% EVOH, respectively. The lower the concentration, the deeper it penetrates into the small peripheral capillary vessels.

The interventional embolization treatment with EVOH was performed applying the previously published plug-and-push-technique [7, 10, 14]: a DMSO compatible microcatheter (usually with a detachable tip, Apollo Onyx Delivery Microcatheter, EV3, Irvine, California, USA) is selectively inserted into the nidus via a feeding artery. The catheter's dead space has to be flushed with DMSO to avoid obstruction by intraluminal polymerization of the EVOH. Before injection, EVOH has to be shaken 10 to 20 minutes by a vibraxer in order to get a homogenous suspension. Then, it is injected very slowly and patiently with 1 ml syringes under fluoroscopy. An EVOH plug generates around the catheter tip and facilitates further antegrade injection. In addition, the plug avoids retrograde flow of EVOH, therefore, providing additional flow toward the nidus and building a coherent cast. The intention of this intervention is to achieve the embolization and closure of as many microfistulae within the AVM as possible. When finished, the microcatheter can be removed by detaching its tip, which is sealed within the plug. Nondetachable catheters have to be withdrawn out of the EVOH plug. The maximal dose of EVOH is 0.5-1.0 ml per kilogram bodyweight in a single procedure. In cases of more extensive lesions, multiple procedures may have to be performed.

**Figure 1:**
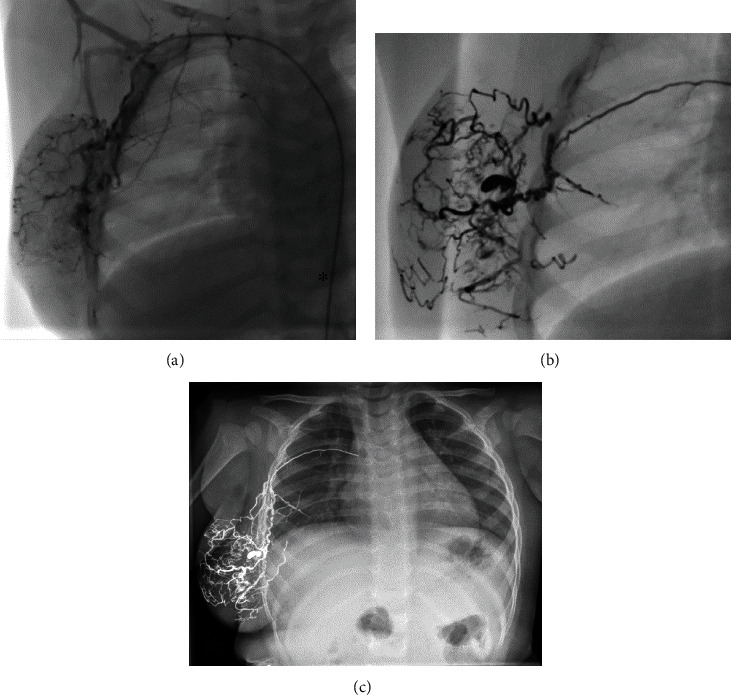
Imaging of the thoracic AVM in patient 2. (a) Angiographical imaging after injection of Solutrast 300® via a transarterial catheter (asterisk) into the feeding arteries deriving from the right subclavian artery. (b) Angiographical imaging after injection of radiopaque Onyx®. (c) Radiographic imaging (X-ray) of the thorax after the last embolization.

In the patients with thoracic AVM (patients 1 and 2), the procedures were conducted under analgosedation with propofol, midazolam, and ketamine. The embolization was performed via the feeding arteries originating from the subclavian and intercostal arteries (see Figure 1 that shows patient 2).

The patient with the pretibial AVM (patient 3) was intubated, ventilated, and in deep analgosedation. The feeding arteries were embolized via the anterior tibial artery. During the first embolization, there was a temporary hypoperfusion caused by extending of the EVOH plug into the anterior tibial artery. Immediate balloon dilatation was necessary with a 2 mm balloon. In a further catheterization, 1 microcoil (diameter 4 mm, Concerto© Helix, Medtronic, Irvine, California, USA) was implanted into a residual AVM artery. There was a transient vascular spasm of this artery during this procedure which was self-limiting within a few minutes. Arterial perfusion of the lower leg quickly normalized in both cases.

**Figure 2:**
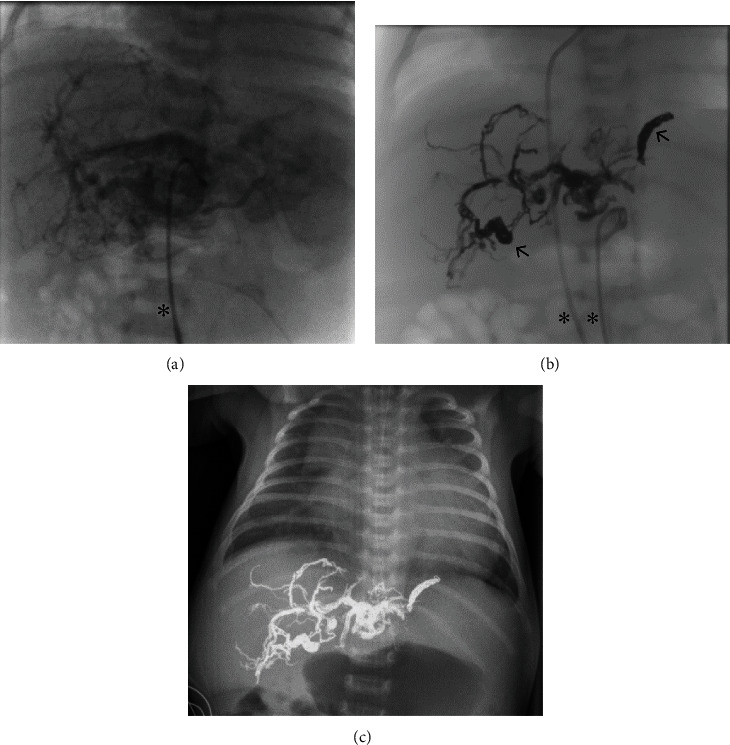
Imaging of the hepatic AVM in patient 4. (a) Angiographical imaging after injection of Solutrast 300® into the feeding arteries via transarterial catheter (asterisk). (b) Angiographical imaging after injection of radiopaque Onyx® and Concerto Helix©-coils (arrows; two transvascular catheters are marked by asterisks). (c) Radiographic imaging (X-ray) of the thorax and upper abdomen after the last embolization, still existing extensive cardiomegaly.

The newborn with the large, prenatally diagnosed hepatic AVM (patient 4) had to be intubated and ventilated immediately postnatally, due to severe high-output cardiac failure and pulmonary hypertension. After interdisciplinary consultation, we decided to embolize the large hepatic AVM with EVOH. Two interventional procedures were necessary within the first 16 days of life. After angiography of the AVM including selective visualization of the feeding arteries via the arteria hepatica communis and the celiac trunc, catheterization and embolization of the feeding arteries were performed. Finally, a right hepatic arterial branch and a left phrenic arterial branch were occluded using 10 detachable microcoils (Concerto© Helix, Medtronic, Irvine, California, USA). The diameters of the coils were 2 mm (3 coils), 4 mm (5 coils), and 5 mm (2 coils), respectively (Figure 2 shows patient 4). A sufficient antegrade perfusion of the liver parenchyma was provided, in contrast to reported cases with complete occlusion of the main hepatic artery.

Embolization methods and results are summarized in Table 2.

### 2.3. Follow-Up

For follow-up, the patients underwent clinical examination, ultrasound, color Doppler imaging, and echocardiography. Blood parameters were obtained in the case of hepatic AVM.

## 3. Results and Discussion

### 3.1. Results

Patients 1 and 2 underwent postinterventional follow-up by clinical examination, ultrasound, and echocardiography after three months, annually thereafter. Due to local hypoperfusion, the AVM transformed by fatty involution and diminished. In both cases, it regressed to a minor soft swelling on the chest wall. Color Doppler ultrasound showed no revascularization. Surgical resection was not necessary. There was an age-appropriate physical resilience. Echocardiography showed a normal cardiac function. The mitral valve competence of patient 1 was significantly improving.

**Figure 3:**
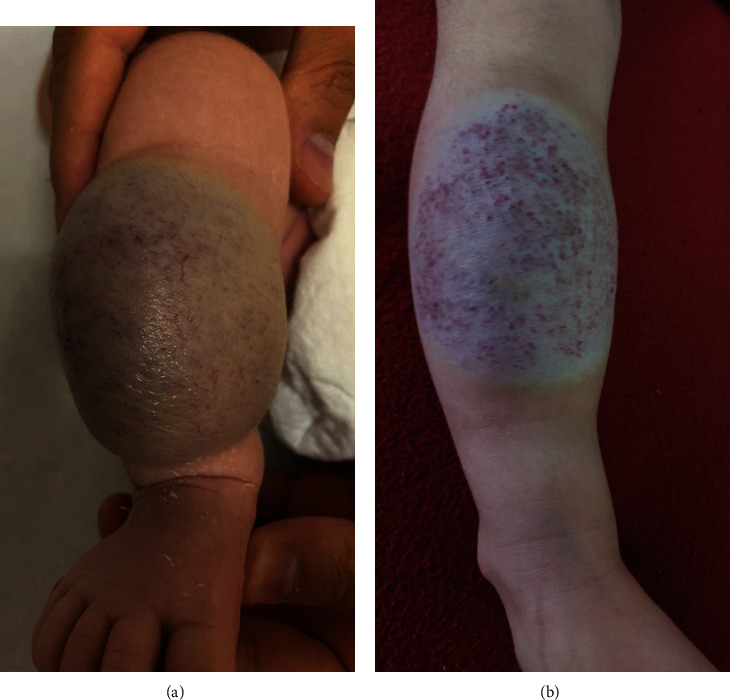
Picture of the tibial AVM in patient 3. (a) Before EVOH embolization. (b) At follow-up.

In patient 3, the AVM partially degenerated with reduction of size, paling, and detumescence within four months after the second embolization (see Figure 3). There was normal leg perfusion and motility. AVM rupture or hemorrhage did not occur. A residual feeding artery was occluded by a microcoil (Concerto© Helix, as mentioned above, diameter 4 mm) in a third intervention.

In these three patients with superficial AVM, slight tattoo effect was seen in terms of minor black skin discoloration.

In patient 4, there was a systemic inflammatory response syndrome (SIRS) with hypotension and increased shock parameters, acidosis, and coagulopathy after the first procedure. At the time of the procedure, the newborn was already in very unstable hemodynamic conditions due to the high-output cardiac failure and suprasystemic pulmonary hypertension. Postinterventionally, intensive care therapy was needed with administration of catecholamines, buffering, and transfusion of fresh frozen plasma and thrombocytes. Weaning from ventilation was feasible on day seven. Before the second procedure, the newborn was electively reintubated and ventilated, and glucocorticoids were administered to avoid possible allergic or hyperinflammatory reaction. At the end of the intervention, electrical cardioversion was necessary because of atrial flutter. SIRS did not recur. The child remained stable and was extubated on day 3 after intervention. The mean pulmonary pressure immediately decreased from 48 to 36 mmHg during the procedure and normalized within the next 3 weeks. Hepatic, renal, and cardiac parameters normalized as well (see Table 3). Within one year after the embolization, all liver parameters were normalized. Several follow-up studies by color Doppler revealed no revascularization of the hepatic AVM.

As a side effect in all patients, there was an uncomfortable smell regressing within about 48 hours, typically caused by the sulfur-containing DMSO.

## 4. Discussion

Embolization is the first-line therapy of AVM. In the last years, liquid embolic agents have gained in importance. There are several published data considering EVOH as suitable for embolization therapy: apart from AVM, EVOH is applied in the treatment of different vascular entities such as gastrointestinal or bronchial hemorrhage [15], endoleaks [16, 17], and central nervous vascular malformations. It can also be used as an adjuvant therapy after surgical AVM resection [18].

Because AVM belong to the group of high-flow malformations, they are attended by high morbidity. Primarily hepatic AVM are associated with a poor prognosis and high mortality, because of severe organ manifestations such as high-output cardiac failure, pulmonary hypertension, and hepatic failure shortly after birth [19, 20]. Due to the critical cardio-respiratory condition immediately after birth, it is challenging to treat hepatic AVM by invasive interventional or surgical procedures. In other reported cases, coil embolization of the main common liver artery [19], extended surgical resection, or liver transplantation are considered as a therapeutic option of large hepatic AVM. Although there are some studies with good clinical results and low risk for adverse effects with EVOH embolization in children [13], there are still few published reports regarding young children, especially infants and newborns. Also, there are very few reports on treatment of neonatal hepatic AVM with EVOH [21, 22].

In comparison to the published cases of Alexander et al. [19] and Hazebroek et al. [23], we were able to preserve the arterial supply of the liver. We prevented the common hepatic artery from total occlusion by diffuse EVOH embolization of the feeding arteries and only selective coil embolizations of branches of hepatic arteries. This may explain the complete normalization of the liver function during follow-up.

Surgical resection of hepatic AVM bears a high risk for hemorrhage [20], which could be avoided as well.

There are remarkable advantages of Onyx© compared to other liquid agents [14]:

- Radiopacity of EVOH due to added tantalum powder allows a good controllability during fluoroscopically guided injection
- Three different available EVOH concentrations feature a variable penetration depth into the AVM nidus. Thus, an extensive occlusion of the malformation is possible
- By polymerizing and building a coherent cast, EVOH reduces the risk of nontarget embolization by detaching particles

There may rarely be severe adverse effects of EVOH treatment such as nontarget embolization with resulting organ ischemia or asystole [24]. Another possible disadvantage is a long procedural duration associated with high radiation exposure, because it has to be injected very slowly and in fractionated procedures.

Especially extremity arteries of young children are small and therefore damageable. Vascular spasms or injuries (dissection, occlusion) can result from extended interventional procedures.

In cases of subcutaneous AVM, there can be a dark discoloration due to superficially injected EVOH. This tattoo effect is caused by the containing black tantalum. The discoloring usually vanishes within some months.

DMSO (most commonly used for cryopreservation of stem cells) is the essential solvent of EVOH to avoid its premature precipitation. It causes an intensive garlic-like smell that volatilizes within 48 to 72 hours after injection. Cardiovascular adverse effects of intravenously administered DMSO can appear in 1-14% [25]. Arterial application of DMSO is always painful, so analgesic treatment during the procedure is necessary.

We performed 8 transcatheter embolizations in four consecutive children with an age range of 3 days to 3 years with considerable success. In three cases, mechanical occlusive devices (coils) were additionally implanted.

Three children had subcutaneous AVM, one had an intrahepatic AVM. In both cases of thoracic wall AVM, liquid embolization with EVOH resulted in complete involution without any severe complication. The tibial AVM could be diminished, restriction of motility or hypoperfusion of the leg as well as rupture and critical bleeding could be avoided. The typical garlic-like smell and slight tattoo effects were the only temporary side effects.

Significant complication was noted in the neonate with the hepatic AVM. The child was in a critical general condition immediately after birth, high-output cardiac failure, and multiorgan dysfunction already existed preinterventionally. Whether the postintervention reaction was, at least partially, related to the DMSO or Onyx© is unclear. Nevertheless, the child recovered after the second procedure with intensive care management, which resulted in a normalized hepatic and cardiac function in long-term follow-up. Extended hepatic surgery and liver transplantation could be avoided.

In summary, our case series confirms previously published data considering EVOH as suitable for treatment of AVM. It is possible to probe deep into the nidus with microcatheters so that it can be occluded widely and persistently. As well on the basis of our experience, exclusive occlusion of main AVM vessels is not a promising therapy. Beyond that, we showed its feasibility in infants as well as in neonates within the first days of life. Catheter size and small peripheral vessels frequently limit interventional procedures. Using microcatheters and guiding wires adapted from the neuroradiology, AVM embolizations are possible via transarterial catheters with small lumina (4 Fr) appropriate for small femoral vessels and thus for patients of the above age group. In contrast to still performed procedures in cases of hepatic AVM, surgical ligation of main hepatic arteries or liver resection can be avoided by this minimally invasive technique. It is thus possible to spare not affected liver tissue and to achieve normalization of biochemical hepatic markers and of the liver function.

We also demonstrated its practicability in cases of emergency interventions in critically ill children suffering from high-output cardiac failure. Postinterventional cardiac reconvalescence occurred.

Furthermore, rupture of superficial AVM attended by acute hemorrhage could be prevented.

The main limitation of our study is the limited number of patients included which can be attributed to the low incidence of AVM as well as to the rare indication for interventional therapy in the first three years of life.

## 5. Conclusion

Percutaneous embolization therapy with EVOH can be performed safely and effectively in the treatment of peripheral AVM in neonates and infants. An additional occlusion with mechanical devices such as detachable microcoils may be helpful. Selective vessel and nidus embolization in neonatal hepatic AVM can prevent acute heart failure; chronic hepatic and other organ dysfunction or even liver transplantation can be avoided. Further follow-up studies with larger a population are necessary to confirm our results.

## Figures and Tables

**Table 1:** Patients' characteristics.

Patient (no.)	Gender	Age at intervention(s)	Body weight (kg)	Localization of the AVM	Symptoms	Schobinger stage
1	f	3 years	12.6	Chest	Heart failure	IV
2	m	14, 16, and 24 months	11.4, 12.0, and 13.5	Chest	Acute AVM hemorrhage	III
4	f	4 months and 8 months	6.6 and 9.7	Right tibia	Increasing size	II
3	m	3 and 16 days	3.4 and 3.5	Liver	Heart failure, pulmonary hypertension	IV

**Table 2:** Embolization method and results.

Patient (no.)	No. of embolization sessions (*n*)	Onyx© concentration/dose (ml)	Additional coils (no. (*n*) and product)	Complications	Follow-up (months)	Result
1	1	18/3 ml	5 Cook©-coils	No	56	Complete AVM involution, normalized cardiac function
2	3	(1) 18/6 ml (2) 18/2.5 ml (3)18/3.2 ml	None	(1) No (2) No (3) No	24	Complete AVM involution
3	2	(1) 18/0.05 ml (2) 18/0.1 ml	1 Concerto Helix©-coil	Transient arterial obstruction	12	Partial AVM involution
4	2	(1) 18/1.5 ml and 34/1.5 ml (2) 18/1.5 ml and 34/1.5 ml	10 Concerto Helix©-coils	(1) SIRS (2) Atrial flutter	11	Complete AVM involution, normalized cardiac and hepatic function, normalized pulmonary pressure

**Table 3:** Laboratory summary of patient 4 (hepatic AVM).

Parameter (normal value)	Before 1. Embolization (peak value)	After 1. Embolization (peak value)	After 2. Embolization (peak value)	At follow-up
ALAT (1-25 U/l)	41	524	28	18
ASAT (10-50 U/l)	96	1,603	41	36
NH_3_ (<102 *μ*g/dl)	81	73	110	54
nt-proBNP (<62.9 pg/ml)	29,334	152,238	14,009	173
Creatinine (0.17-0.42 mg/dl)	0.92	0.99	0.25	0.23

Abbreviations: ALAT: alanine aminotransferase; ASAT: aspartate aminotransferase; NH_3_: ammonia; nt-proBNP: N-terminal pro-brain natriuretic peptide.

## Data Availability

The data underlying the present study are available on request (corresponding author).

## Abbreviations

AVM: Arteriovenous malformation
DMSO: Dimethyl sulfoxide
EVOH: Ethylene-vinyl alcohol copolymer
Fr: French (1 French = 1/3 mm)
ISSVA: International Society for the Study of Vascular Anomalies
SIRS: Systemic inflammatory response syndrome.
